# Evaluation of circulating tumor cell gene expression profiles before and following combination immunotherapy for advanced prostate cancer

**DOI:** 10.1186/2051-1426-2-S3-P257

**Published:** 2014-11-06

**Authors:** Sachin Puri, Jing Han, Bharat H Joshi, Daniel Healy, Janet Siebert, Christopher Paustian, Christopher Dubay, Keith S Bahjat, Brendan Curti, Walter J Urba, Bernard A Fox

**Affiliations:** 1 Robert W. Franz Cancer Research Center, Earle A. Chiles Research Institute, Providence Cancer Center, Portland, OR, United States; 2 Division of Cellular and Gene Therapies, Center for Biologics Evaluation and Research, FDA, Bethesda, MD, United States; 3 CytoAnalytics, Denver, CO, United States; 4 Molecular Microbiology and Immunology, OHSU, Portland, OR, Portland, OR, United States

## 

The Phase I/II clinical trial randomized patients to GVAX immunotherapy (two prostate cancer cell lines that secrete GM-CSF, Cell Genesys Inc.) alone or in combination with non-myeloablative chemotherapy and adoptive transfer of PBMC. Pre and week 11 aphereses were obtained to elutriate monocytes and cryopreserve PBMC for immune monitoring. We used ProtoArrays (8,217 proteins spotted arrays, Invitrogen) to evaluate the week 11 post treatment antibody responses of 11 patients who completed this IRB-approved DAMD-funded clinical trial for men with androgen-independent prostate cancer. Analysis of pre and week 11 sera identified increased antibody responses to a number of proteins and the patients with an increase in PSA-doubling time (PSA-DT) exhibited a strong response to a significantly (p < 0.05) larger number of proteins (mean = 60) than patients whose PSA-DT decreased (mean = 22). Next we sought to determine whether the genes targeted by the strong Ab responses were expressed by the patient's tumor. Since no post treatment tumor biopsies were available, a fluorescence-activated cell sorting (FACS) method was developed to isolate circulating tumor cells from cryopreserved blood samples. Aphereses products were thawed, stained with anti-EpCAM, anti-CD45, anti-CD4, and anti-CD8 antibodies. EpCAM +ve and CD4, CD8, CD45 -ve cells were sorted as CTC. RNA was isolated from all CTC and amplified. Gene expression profiling was performed using 35K oligonucleotide arrays. Amplified RNA from CTCs were hybridized with Cy5 and amplified normal prostate RNA were hybridized with Cy3 Dye. Data was analysed using madb (BIMAS/CBEL/CIT, NCI). Gene expression profile was compared between pre and post treatment CTCs. CTC expressed 1296-2369 genes at >1.5 fold compared to a normal prostate tissue control. Current efforts are aimed at evaluating whether targets of the antibody response were contained in the oligonucleotide array and whether proteins for genes that decreased in intensity were contained on the protein array. The methods we describe may help assess whether a given treatment is inducing strong immune response and whether this immune response is sculpting the repertoire of antigens expressed by the tumor. Such tests are currently not available and development of such a test may aid the development and refinement of the novel combination immunotherapy strategies.

## Support

The Prostate Cancer Foundation, DAMD PC02009, Steve and Cindy Harder, Nancy and Wes Lematta, Robert W. and Elsie Franz, Lynn and Jack Loacker and The Chiles foundation.

## Consent

Written informed consent was obtained from the patient for publication of this abstract and any accompanying images. A copy of the written consent is available for review by the Editor of this journal.

